# Fire Appliances for Use in Hospitals

**Published:** 1909-06-05

**Authors:** 


### HOSPITAL ADMINISTRATION.

### CONSTRUCTION AND ECONOMICS.

### FIRE APPLIANCES FOR USE IN HOSPITALS.

#### II.—AUTOMATIC FIRE EXTINGUISHERS AND LIFE-SAVING APPARATUS.

In addition to those described in the previous article, another class of apparatus has been gradually developed, designed to come into operation automatically should the temperature at any part of a building rise above a certain figure. The appliances were originally worked out for use in the Lancashire cotton mills, but they have gradually been adopted by a number of buildings in which other industries are housed. Flour mills, for instance, have adopted them very largely, and also the large co-operative buildings that are rising in different parts of the country, and in America schools have adopted them. The apparatus is arranged to produce what is practically a shower bath, a fine spray of water, covering a comparatively wide area, in the neighbourhood of the spot where the rise of temperature occurs. The idea underlying the design is that in a great many buildings fires occur at night, or may break out in portions of a building where no one is present at the moment, and get a firm hold of the building before they are discovered. In such cases the rise of temperature opens a valve, through which the spray of water mentioned is forced, drenching everything in the immediate neighbourhood. The apparatus has the double advantage that it comes into operation during that critical early period when it is possible, if ever, to stop a serious fire, and the damage by water, which is often more serious even than that from fire, is confined to the immediate neighbourhood. In the case of hospitals the portions of the building to which the appliances could be adapted would require very careful consideration. But there is a large part of all hospital buildings where they might with advantage be used. The portions of the buildings referred to are those where, under modern conditions, fire is very likely to break out. All modern hospitals have heating plant, usually boilers, furnaces, and various apparatus, in which high temperatures are dealt with, and it is common knowledge that carelessness on the part of those employed sometimes leads to serious fires under similar conditions. The automatic appliances are known as "sprinklers," the Grinnell, made by Messrs. Mather & Platt, of Salford, being the one best known on both sides of the Atlantic. The building to be protected is piped, just as for a gas or water service, but the pipes which are to supply the "sprinklers" are fixed under the ceilings. The system of pipes is connected either to the town supply, to a large tank overhead, to a water service worked by a pump which also comes into operation automatically, or, as it is preferred in some cases in America, to a tank fixed on an elevation at a little distance from the building. The valve is held closed under ordinary conditions by two strips of metal soldered together. The solder can be made to melt at any temperature, the usual figure being 155° F., which gives early warning of danger. On the arrival of that temperature the solder melts, the strip gives way, the valve opens, and the shower-like spray comes into operation, a large alarm bell usually ringing at the same time. A modification of this arrangement is used to protect buildings from fires taking place in neighbouring buildings—another important matter. Pipes are laid on the outsides of the buildings, near the roof, and are fitted with valves, either arranged to come into operation automatically, if the temperature in the neighbourhood rises, or to be operated by hand from some convenient spot. The pipes are connected to the water service, and, when in operation, a water screen is formed which efficiently cuts off sparks, flames, etc.

In the case of hospitals, a more important matter is the question of saving life in case of fire. Most modern hospitals are fitted with iron ladders outside the buildings, through which the inmates of the upper stories can escape, but in certain cases it will be remembered that even these cannot easily be used. Patients could hardly get down them, and in the case of hospitals not so provided, other means are required. The firms who have made a speciality of fire-fighting appliance, Messrs. Merryweather and others, have also worked out apparatus of various forms for rescuing patients, and others on the upper stories. A number of apparatus have been developed, by the aid of rope and canvas. A great deal can be done with a rope by those who know how, if some support can be found for it on the floor from which rescue is to be carried out. Brackets, hooks, and other appliances are arranged to be fixed in each floor, near the different windows, and ropes with pulleys attached, and with chairs, hammocks, what sailors call "bowline on the bight" knots, and other arrangements are made to support patients and others. For those who are able to hold on to a rope, while seated in a chair, the matter of rescue is very simple, provided that a convenient support for the pulley in which the rope runs can be found. Where no other support is available, one or two bedsteads drawn close up to the window, and the rope attached to the pulley taken round them, are usually sufficient. The patient to be rescued is placed in the chair, or in the knot mentioned, which is arranged like a chair, one loop for him to sit on, and another loop under the arms, or in the hammock and is lowered, either from above or below. In another set of apparatus canvas shoots are provided, held to brackets, or other supports, at the window from which rescue is to take place, the lower ends being held as far out from the building as possible by those in the street. The person to be rescued is placed in the shoot at the window, and slides down to the bottom, if everything is properly arranged, without harm. Of course, the utility of all of these apparatus, as well as of the portable fire extinguishing machines described in the previous article, depends upon their being on the spot when wanted, and upon there also being present someone with a cool head who understands the appliance.

